# Supplementary material for: An assessment of non-communicable disease mortality among adults in Eastern Uganda, 2010–2016
Source: PLoS One. 2021 Mar 19;16(3):e0248966. doi: 10.1371/journal.pone.0248966 (PMC7978282; doi:10.1371/journal.pone.0248966)
Supplement: Attachment — Submitted filename: Review for PloS - Natukwatsa et al, 2020.docx [file pone.0248966.s001.docx]

Review for the PLoS article

**Title: An analysis of non-communicable disease mortality among adults in Eastern Uganda, 2010-2016**

1. **Do the title and abstract accurately convey what has been found?**

Yes, title is ok, but I would use the word “assessment” instead of analysis – this is usually reserved for a novel way of analysis e.g., new statistical analysis method

2. **Is the writing acceptable?**

Yes, writing is acceptable.

3. **Are the data sound?**

Yes, steps followed look sound and well documented

4. **Do the figures appear to be genuine, i.e., without evidence of manipulation?**

Yes, figures appear genuine

5. **Does the manuscript adhere to the relevant standards for reporting and data deposition?**

Yes, standards are adhered to

6. **Are the discussion and conclusions well balanced and adequately supported by the data?**

Yes, but discussion is quite light might need some beefing up.

7. **Are limitations of the work clearly stated?**

Yes, study limitations are clearly stated.

8. **Do the authors clearly acknowledge any work upon which they are building, both published and unpublished?**

Yes, this is done well in the discussion perhaps a little more in the introduction.

9. **General Observation**

Overall, it is a well described and interesting paper that adds to the current body of evidence and speaks to the current NCD burden especially NCD mortality within sub-Saharan Africa generally and Uganda specifically.

10. **Minor Essential Revisions**

- Classification of outcomes - indicate how multiple causes of death or multiple conditions present at time of death were handled and death classified in that case e.g., if at death someone had both Cancer and DM or had both DM and accidental death
- Need to reformat the tables to highlight points of interest e.g., bolding or re-aligning or smaller font size for some figures e.g. Table 2 can be better displayed as below

| **2016** | | | **2015** | | | **2014** | | |
| --- | --- | --- | --- | --- | --- | --- | --- | --- |
| Men | Women | **Overall** | Men | Women | **Overall** | Men | Women | **Overall** |
| 11.29 19.68 **16.78** | | | 7.9 21.48 **14.91** | | | 10.15 21.86 **15.02** | | |

- Indicate the AAMR units e.g. is it deaths per 10,000 or per 100,000?
- Built up the discussion nicely to indicate the burden and CV risk factor profile in Iganga-Mayuge - but do but not tie it up to show how this would lead to a high CVD mortality e.g.
  - What are the known mortality trends in the region if any e.g. Eastern Uganda, in Uganda or E. Africa?
  - How do these trends compare with what was found?
  - How do they compare with regional hospital mortality trends, any surprises there??
  - What might account for the anomalies e.g., fewer deaths in females, fewer deaths to DM in 2016 etc..
  - How might these data on mortality be useful going forward, perhaps operationally??

11. **Level of interest**

I think this is a worthwhile study and a timely addition of valuable information to the current body of evidence with regard to NCD mortality in sub-Saharan Africa.

12. **Quality of written English**

Acceptable

13. **Declaration of competing interests**

I declare that I have no competing interests
